# Bleeding risk factors and real-world antithrombotic therapies in elderly patients with atrial fibrillation undergoing percutaneous coronary intervention: a retrospective study

**DOI:** 10.1186/s40780-023-00308-8

**Published:** 2023-12-12

**Authors:** Kanako Fujita, Noriko Kohyama, Miki Sato, Tomokazu Deguchi, Hiroshi Suzuki, Mio Ebato, Mari Kogo

**Affiliations:** 1 Division of Pharmacotherapeutics, Department of Clinical Pharmacy, Showa University School of Pharmacy, 1-5-8 Hatanodai, Shinagawa-ku, Tokyo, 142-8555 Japan; 2 Department of Pharmacy, Showa University Fujigaoka Hospital, Yokohama, Japan; 3 Division of Cardiology, Department of Internal Medicine, Showa University Fujigaoka Hospital, Yokohama, Japan

**Keywords:** Atrial fibrillation, Percutaneous coronary intervention, Aged, Hemorrhage, Risk factor

## Abstract

**Background:**

Bleeding risk factors in elderly patients with atrial fibrillation undergoing percutaneous coronary intervention (PCI) are unclear and data on the use of antithrombotic drugs are lacking. We investigated the bleeding risk factors in elderly patients with atrial fibrillation undergoing PCI to help optimize antithrombotic therapy according to bleeding risk. We also investigated the association between the actual use of antithrombotic therapy and bleeding events.

**Methods:**

A retrospective cohort study was conducted on 134 elderly patients with atrial fibrillation who underwent primary PCI at the Department of Cardiology, Showa University Fujigaoka Hospital. The endpoint was a bleeding event within 1-year. Bleeding risk factors were identified using multivariate analysis. The association between the number of antithrombotics and bleeding events was evaluated using the chi-squared test.

**Results:**

The mean age of the patients was 76.0 ± 6.2 years. Bleeding events occurred in 41 (30.6%) patients. Age > 80 years (odds ratio [OR]: 2.54, 95% confidence interval [CI]: 1.10–5.85), multivessel disease (OR: 2.76, 95% CI: 1.22–6.23), and history of surgery (OR: 3.03, 95% CI: 1.14–8.06) were identified as bleeding risk factors. The proportion of patients receiving triple therapy was significantly higher in the bleeding group compared to the non-bleeding group (70.7% vs. 27.5%, *p* < 0.001).

**Conclusions:**

Age > 80, multivessel disease, and history of surgery were found to be risk factors for bleeding in elderly patients with atrial fibrillation undergoing PCI. In addition, dual therapy after PCI in elderly patients at high risk of bleeding should be considered to avoid bleeding events.

**Supplementary Information:**

The online version contains supplementary material available at 10.1186/s40780-023-00308-8.

## Background

Atrial fibrillation (AF) is an irregular heart rhythm caused by chaotic electrical impulses from the atria that are conducted through the ventricles. The Global Burden of Disease Project reported a global prevalence of approximately 40.6 million persons with AF in 2017 [1]. AF is a risk factor for cardiogenic brain embolism that requires long-term oral anticoagulation if the risk of embolic events is high [2].

Approximately 20–30% of patients with AF have concomitant ischemic heart diseases, such as angina or myocardial infarction [3]. Percutaneous coronary intervention (PCI) is widely used for revascularization in ischemic heart disease. Patients undergoing PCI are administered dual antiplatelet therapy with a P2Y12 inhibitor and aspirin to prevent stent thrombosis and recurrent myocardial infarction [4]. The widespread use of second-generation drug-eluting stents and hydroxymethylglutaryl-coenzyme A (HMG-CoA) inhibitors has significantly reduced the incidence rate of stent thrombosis after PCI to approximately 1% [5].

In contrast, the rate of bleeding events associated with the use of anticoagulants in patients with AF is 10–15%; moreover, the use of both anticoagulants and antiplatelet agents after PCI increases the risk of bleeding in patients with AF compared with anticoagulation alone [6, 7], and even more so in elderly patients [8]. Therefore, in elderly patients with AF undergoing PCI, the high risk of bleeding due to concomitant antithrombotic use and age is a therapeutic challenge. As the world’s population ages, the number of very elderly patients with AF undergoing PCI is increasing [9]. As bleeding complications after PCI are strongly associated with mortality [10], it is important to predict the risk of bleeding in elderly patients with AF undergoing PCI.

Several risk factors for bleeding have been reported in patients undergoing PCI or with AF. Major factors include reduced renal function, anemia, history of bleeding, history of surgery or trauma, concomitant antithrombotic drugs, and older age [11, 12]. However, the risk factors for bleeding in elderly patients with AF undergoing PCI are unclear. The identification of bleeding risk factors in these patients may help to plan treatment, including setting the intensity and duration of antiplatelet medication.

Current guidelines recommend shortening the duration of triple therapy [oral anticoagulant (OAC) + dual antiplatelet therapy (DAPT)], followed by dual therapy (OAC + antiplatelet agent alone) for up to 12 months after PCI if the risk of bleeding is high [13]. However, there is a lack of data on antithrombotic therapy in elderly patients with AF undergoing PCI.

We aimed to identify the risk factors of bleeding events in elderly patients with AF undergoing PCI to help optimize antithrombotic therapy according to bleeding risk. We also aimed to clarify the association between the actual use of antithrombotic therapy and bleeding events.

## Methods

### Ethics approval

This study was approved by the Ethics Committee of the Showa University School of Pharmacy, Japan (Approval No. 379 on June 11, 2020). This study was conducted in accordance with the principles of the Declaration of Helsinki.

### Study population

Out of the 2,608 patients who underwent primary PCI, this retrospective cohort study enrolled 201 patients aged 65 years or older with AF who were admitted to Showa University Fujigaoka Hospital from April 1, 2009 to March 31, 2020 and were started on antiplatelet therapy. The exclusion criteria were as follows: no anticoagulant use during hospitalization; upcoming surgery; history of valve replacement; and aneurysms. Patients who could not participate in follow-up for 1 year after the index PCI and those with bleeding events related to the index PCI were also excluded. Sixty-seven patients were excluded and 134 patients were included in the analysis (Additional file: Supplementary material 1).

### Data collection of patient characteristics

Data were collected from the medical records of participants. Baseline patient characteristics included age, sex, body weight, body mass index, smoking history, alcohol consumption history, heart rate, systolic blood pressure, diastolic blood pressure, history of surgery, history of bleeding, history of coronary artery bypass grafting, and concomitant disease or medical history. Baseline lesion and interventional characteristics included the number of target lesions at index PCI, lesion site, chronic total occlusion, multivessel disease, stent type, and clinical presentation. Laboratory data included white blood cell count, albumin, hemoglobin, platelets, aspartate aminotransferase, alanine aminotransferase, estimated glomerular filtration rate (eGFR), creatinine clearance (CrCl), creatinine kinase, C-reactive protein, brain natriuretic peptide, prothrombin time-international normalized ratio, glycosylated hemoglobin (HbA1c), high-density lipoprotein cholesterol, low-density lipoprotein cholesterol, triglycerides, and left ventricular ejection fraction.

Patient information at discharge included duration of hospital stay and medications, such as type and dose of oral anticoagulants, number of antithrombotic agents, proton pump inhibitors, HMG-CoA inhibitors, nonsteroidal anti-inflammatory drugs, eicosapentaenoic acid, P-glycoprotein inhibitors, and long-term use of glucocorticoids.

### Definitions

Participants who were 65 years or older were considered elderly, according to the World Health Organization classification of elderly individuals. A history of surgery was defined as any surgery performed under general anesthesia, endoscopic surgery, or catheter ablation within 1 year before PCI. A history of bleeding was defined according to the Bleeding Academic Research Consortium (BARC) class ≥ 2 [14]. Anemia was defined as a history of anemia or hemoglobin level < 11 g/dL [15]. Hypertension was defined as casual blood pressure ≥ 140/90 mmHg or the current use of antihypertensive drugs. Dyslipidemia was defined as the use of a lipid-lowering agent or casual low-density lipoprotein cholesterol level > 140 mg/dL, high-density lipoprotein cholesterol level < 40 mg/dL, or triglyceride level > 150 mg/dL. Diabetes was defined as treatment with insulin or oral hypoglycemic agent, casual plasma glucose level > 200 mg/dL, or HbA1c > 6.5%.

### Cut-off considerations

eGFR was considered with cut-off values of 15, 30, 45, and 60 mL/min/1.73 m², in accordance with the severity classification outlined in the CKD guidelines. The odds ratio (OR) demonstrated a linear increase when eGFR was stratified into three groups based on the cut-off values of < 30, 30–45, and > 45 mL/min/1.73 m². Additionally, renal function was assessed based on CrCl. The cut-off values for CrCl were < 30, 30–50, and > 50 mL/min, which are the selection criteria for determining the appropriate anticoagulant dose. The cut-off value for age was set at 80 years based on the receiver operating characteristic curve (Additional file: Supplementary material 2).

### Clinical endpoints and follow-up

The primary endpoints were defined as BARC class 2, 3a, 3b, 3c, or 5 bleeding events within 1 year after the index PCI, while the secondary endpoints were BARC 3 or 5 major bleeding events within one year after the index PCI [14]. The first bleeding event was collected if multiple bleeding events occurred during 1 year of follow-up.

### Status of the use of antithrombotic drugs

Anticoagulant and antiplatelet status was assessed after PCI and monitored during 1 year of follow-up. Antiplatelet drugs included aspirin, clopidogrel, and prasugrel. For each year of PCI, the proportion of patients continuing triple antithrombotic therapy for 1, 3, 6, and 12 months after discharge was calculated.

We defined OAC as warfarin, dabigatran, rivaroxaban, apixaban, and edoxaban. For direct oral anticoagulants (DOACs) administration, “standard dose” and “low-dose” were defined as administration according to the standard or low-dose regimens, respectively. Dose selection of each DOAC was evaluated based on the manufacturer labeling recommendations in Japan. The following low-dose regimens were considered appropriate: dabigatran 110 mg (twice daily) for patients aged ≥ 70 years, with a CrCl of 30–50 mL/min or a history of bleeding; rivaroxaban 10 mg (once daily) for patients with a CrCl of 15–50 mL/min; apixaban 2.5 mg (twice daily) for patients with any two of the following characteristics: age ≥ 80 years, with a body weight of ≤ 60 kg, and a serum creatinine level of ≥ 1.5 mg/dL; and edoxaban 30 mg (once daily) for patients with a CrCl of 15–50 mL/min or a body weight of ≤ 60 kg.

Underdosing (off-label low-dose) therapy was defined as the use of a low-dose of DOACs despite meeting the standard dose criteria. Overdosing (off-label standard dose) therapy was defined as the use of a standard dose of DOACs despite meeting the low-dose regimen criteria. The number of antithrombotic agents was investigated at the time of bleeding in the bleeding event group and at the end of observation in the without-bleeding event group. Triple therapy was defined as the combination of an anticoagulant and DAPT, while dual therapy was defined as the combination of an anticoagulant and a single antiplatelet drug.

### Statistical analysis

#### Sample size

We calculated that the required sample size was 105 patients to detect an OR of 2.0 with 80% power at a significance level of 0.05, using an equivalence test of the hypotheses and assuming the bleeding event rate to be 20%. Therefore, the sample size in this study was sufficient to reach a valid conclusion.

#### Univariate and multivariate analyses

Continuous variables were presented as mean ± standard deviation. Categorical variables were presented as the number of persons with frequency. Fisher’s exact test or chi-square test was used to compare categorical variables of patients with and without bleeding events. Significant variables extracted using the univariate analysis with less than 10% missing values were entered into the multivariate analysis. Then, a multivariate logistic regression analysis was performed. A stepwise selection method was used to identify factors independently associated with bleeding events and major bleeding events after PCI.

Additionally, a subgroup analysis was conducted to identify factors independently associated with bleeding events after PCI in two specific populations: (1) individuals receiving VKA at discharge and (2) individuals receiving DOACs at discharge. *p* < 0.05 was considered statistically significant. All statistical analyses were performed using SPSS software version 25 (IBM Corp., Armonk, NY, USA).

#### Development and evaluation of a bleeding risk model

A formula for predicting the bleeding risk was developed using logistic regression analysis. The analysis was performed by adding three risk factors for bleeding events - eGFR [11], anemia [11], and hypertension [16] - as covariates to statistically significant factors determined by the multivariate analysis (*p* < 0.05). C-statistics of established bleeding risk factors were calculated, and factors with high C-statistics were selected as covariates (Additional file: Supplementary material 3). The accuracy of each model formula was then compared using the C-statistic, and the model formula with the highest C-statistic was selected as the final model (Additional file: Supplementary material 4).

Furthermore, a risk model was developed using the prediction formula for the bleeding risk. The OR for each factor in the final model formula was divided by the smallest OR among the factors and approximated to the nearest integer. Each factor was then assigned a score of 2 points if the calculation result was ≥ 2, and 1 point if it was < 2. The accuracy of the risk model was evaluated by calculating the sensitivity, specificity, positive predictive value, and negative predictive value. The total score for each patient was calculated by summing the scores of each factor. Patients were divided into three groups based on their scores, and bleeding event rates were compared among the three groups using chi-square tests.

#### Association between antithrombotic use and bleeding events

The associations of triple therapy duration with bleeding events, DOAC dose at discharge with bleeding events, and the number of anticoagulants with bleeding events were each examined using the chi-square test. In the triple therapy and dual therapy, the bleeding rates between the developed risk models were compared using the chi-square test. As the number of antithrombotic agents can potentially vary over time, the study delved into exploring the relationship between the number of such agents at the time of bleeding and the occurrence of bleeding events. The relationship between the number of antithrombotic agents at the time of bleeding or at the end of observation and bleeding events was investigated using the chi-square test and logistic regression analysis.

## Results

### Patient characteristics

The baseline patient characteristics, PCI profile, and discharge medications are summarized in Table 1. The mean age was 76 years (± 6.2 years) and 111 participants (82.8%) were male. Twenty-two (16.4%) patients had a history of surgery, including gastrointestinal surgery (n = 10), surgery related to malignancy (n = 7), orthopedic surgery (n = 4), thoracotomy for cardiac rupture (n = 1), and other endoscopic surgery (n = 3). At discharge, 130 patients (97.0%) were on triple therapy, while four patients (3.0%) were on dual therapy.

**Table 1:** Baseline patient characteristics, PCI profile, and discharge medications

Variables	mean ± SD, n (%), median (MIN–MAX) n = 134		Missing value (%)	
Age (years)	76.0	± 6.2	0.0	
Sex (male)	111	(82.8)	0.0	
Body weight (kg)	60.53	± 10.69	0.0	
Body mass index (kg/m^2^)	22.83	± 3.00	0.0	
Smoking history	83	(61.9)	0.0	
Alcohol consumption history	62	(46.6)	0.7	
Heart rate (times/min)	73.9	± 19.0	0.0	
Systolic blood pressure (mmHg)	120.5	± 22.0	0.0	
Diastolic blood pressure (mmHg)	68.5	± 13.8	0.0	
History of surgery	22	(16.4)	0.0	
History of bleeding	19	(14.2)	0.0	
History of CABG	6	(4.5)	0.0	
Concomitant disease or medical history				
Hypertension	101	(75.4)	0.0	
Dyslipidemia	71	(53.0)	0.0	
Diabetes mellitus	46	(34.3)	0.0	
Cerebral infarction	25	(18.7)	0.0	
Myocardial infarction	16	(11.9)	0.0	
Heart failure	55	(41.0)	0.0	
Malignancy	25	(18.7)	0.0	
Dialysis	5	(3.7)	0.0	
Liver disease	8	(6.0)	0.0	
Anemia	30	(22.4)	0.0	
Gastrointestinal disease	30	(22.4)	0.0	
Peripheral arterial disease	16	(11.9)	0.0	
PCI profile				
Number of target lesions	1.4	± 0.6	0.0	
Lesion site			0.0	
Right coronary artery	31	(23.1)	0.0	
Left main trunk	5	(3.7)	0.0	
Left anterior descending coronary artery	76	(56.7)	0.0	
Left circumflex coronary artery	22	(16.4)	0.0	
Chronic total occlusion	33	(24.6)	0.0	
Multivessel disease	69	(51.5)	0.0	
Stent type			0.0	
Bare metal stent	24	(17.9)	0.0	
Drug eluting stent	110	(82.1)	0.0	
Clinical presentation			0.0	
Acute coronary syndrome	63	(47.0)	0.0	
Stable coronary artery diseases	71	(53.0)	0.0	
Laboratory data				
Albumin (g/dL)	3.7	± 0.5	0.0	
White blood cell (/µL)	6400.0	(3300–19950)	0.0	
Hemoglobin (g/dL)	12.80	± 1.97	0.0	
Platelet (10^4^/µL)	19.84	± 6.46	0.0	
Aspartate aminotransferase (U/L)	27.5	(13–501)	0.0	
Alanine aminotransferase (U/L)	22.0	(4–449)	0.0	
eGFR (mL/min/1.73 m^2^)	56.61	± 19.22	0.0	
Creatinine kinase (IU/L)	94.0	(17–5261)	0.7	
C-reactive protein (mg/L)	0.34	(0.03–17.00)	12.7	
Brain natriuretic peptide (pg/mL)	246.80	(14.4–3520.2)	6.7	
PT-INR	1.23	± 0.35	9.0	
Hemoglobin-A1c (NGSP %)	6.42	± 1.07	21.6	
High-density lipoprotein (mg/dL)	50.4	± 14.6	9.7	
Low-density lipoprotein (mg/dL)	103.4	± 29.1	12.7	
Triglyceride (mg/dL)	91.7	± 56.1	11.9	
Left ventricular ejection fraction (%)	50.5	± 13.7	3.0	
Duration of hospital stay (days)	12.0	(2–83)	0.0	
Medication at discharge				
Type of oral anticoagulants			0.0	
Vitamin K antagonist	54	(40.3)	0.0	
Direct oral anticoagulant	80	(59.7)	0.0	
Proton pump inhibitors	114	(85.1)	0.0	
HMG-CoA reductase inhibitors	86	(64.2)	0.0	
Nonsteroidal anti-inflammatory drugs	10	(7.5)	0.0	
Eicosapentaenoic acids	2	(1.5)	0.0	
P-glycoprotein inhibitors	6	(4.5)	0.0	
Long-term use of glucocorticoids	2	(1.5)	0.0	

PCI, percutaneous coronary intervention; SD, standard deviation; eGFR, estimated glomerular filtration rate; PT INR, prothrombin time international normalized ratio; NGSP, national glycohemoglobin standardization program

### Endpoints

Bleeding events classified as BARC class ≥ 2 occurred in 41 (30.6%) patients. The median time to the bleeding event was 70 (range: 3–363) days. The proportion of each bleeding event according to BARC criteria is as follows: BARC 2: 25 (61.0%); BARC 3a: 10 (24.4%); BARC 3b: 3 (7.3%); BARC 3c: 1 (2.4%); BARC 5: 2 (4.9%) (Additional file: Supplementary material 5). The location of the bleeding event is also shown in Additional file: Supplementary material 5.

### Univariate and multivariate analyses

#### Risk factors for bleeding events

Age > 80 years [OR: 2.536, 95% confidence interval (CI): 1.099–5.853; *p* = 0.029], multivessel disease (OR: 2.761, 95% CI: 1.223–6.231; *p* = 0.014), and history of surgery (OR: 3.031, 95% CI: 1.141–8.055; *p* = 0.026) were identified as independent risk factors of bleeding events after PCI in elderly patients with AF (Table 2).

**Table 2:** Bleeding risk factors in elderly patients with AF undergoing PCI

Variables	With bleeding events (n = 41)		Without bleeding events (n = 93)		Univariate analysis		Multivariate analysis^†^ (stepwise selection method)	
					*p*-value		OR (95% CI)	*p*-value
	n (%)		n (%)					
Age > 80 years	17	(41.5)	21	(22.6)	0.025^a)*^		2.536 (1.099–5.853)	0.029^*^
Sex, male	32	(78.0)	79	(84.9)	0.329^a)^			
Body mass index < 20 (kg/m^2^)	11	(26.8)	15	(16.1)	0.149^a)^			
Smoking history	30	(73.2)	53	(57.0)	0.075^a)^			
Alcohol consumption history	18	(45.0)	44	(47.3)	0.806^a)^			
Systolic BP ≥ 140 (mmHg)	6	(14.6)	21	(22.6)	0.291^a)^			
Diastolic BP ≥ 90 (mmHg)	4	(9.8)	7	(7.5)	0.736^b)^			
History of surgery	12	(29.3)	10	(10.8)	0.008^a)*^		3.031 (1.141–8.055)	0.026^*^
History of bleeding	10	(24.4)	9	(9.7)	0.024^a)^			
History of CABG	3	(7.3)	3	(3.2)	0.370^b)^			
Concomitant disease or medical history								
Hypertension	35	(85.4)	66	(71.0)	0.075^a)^			
Dyslipidemia	26	(63.4)	45	(48.4)	0.156^a)^			
Diabetes mellitus	15	(36.6)	31	(33.3)	0.715^a)^			
Cerebral infarction	7	(17.1)	18	(19.4)	0.755^a)^			
Myocardial infection	5	(12.2)	11	(11.8)	1.000^b)^			
Heart failure	19	(46.3)	36	(38.7)	0.408^a)^			
Malignancy	10	(24.4)	15	(16.1)	0.258^a)^			
Dialysis	2	(4.9)	3	(3.2)	0.642^b)^			
Liver disease	3	(7.3)	5	(5.4)	0.700^b)^			
Anemia	14	(34.1)	16	(17.2)	0.030^a)*^			
Gastrointestinal disease	13	(31.7)	17	(18.3)	0.086^a)^			
Peripheral arterial disease	5	(12.2)	11	(11.8)	1.000^b)^			
PCI profile								
Number of target lesions ≥ 2	15	(36.6)	33	(35.5)	0.902^a)^			
Lesion site					0.866^b)^			
RCA	10	(24.4)	21	(22.6)				
LMT	1	(2.4)	4	(4.3)				
LAD	22	(53.7)	54	(58.1)				
LCX	8	(19.5)	14	(15.1)				
Chronic total occlusion	11	(26.8)	22	(23.7)	0.694^a)^			
Multivessel disease	28	(68.3)	41	(44.1)	0.010^a)*^		2.761 (1.223–6.231)	0.014^*^
Stent type					0.418^a)^			
BMS	9	(22.0)	15	(16.1)				
DES	32	(78.0)	78	(83.9)				
Clinical presentation					0.306^a)^			
ACS	22	(53.7)	41	(44.1)				
stable CAD	19	(46.3)	52	(55.9)				
Laboratory data								
Albumin < 3.5 (g/dL)	14	(34.1)	29	(31.2)	0.735^a)^			
White blood cell ≥ 10,000 (/µL)	9	(22.0)	10	(10.8)	0.087^a)^			
Hemoglobin < 11 (g/dL)	9	(22.0)	15	(16.1)	0.418^a)^			
Platelet < 10 (10^4^/µL)	2	(4.9)	1	(1.1)	0.222^b)^			
AST > 50 (U/L)	13	(31.7)	19	(20.4)	0.158^a)^			
ALT > 50 (U/L)	6	(14.6)	13	(14.0)	0.920^a)^			
eGFR (mL/min/1.73m^2^)					0.086^a)^			
< 30	6	(14.6)	4	(4.3)				
30–45	7	(17.1)	13	(14.0)				
> 45	28	(68.3)	76	(81.7)				
CrCl (mL/min)					0.124^a)^			
< 30	8	(19.5)	7	(7.5)				
30–50	13	(31.7)	36	(38.7)				
> 50	20	(48.8)	50	(53.8)				
Creatinine kinase > 230 (IU/L)	8	(20.0)	23	(24.7)	0.554^a)^			
C-reactive protein > 0.3 (mg/L)	21	(56.8)	40	(50.0)	0.496^a)^			
BNP ≥ 100 (pg/mL)	28	(73.7)	65	(74.7)	0.904^a)^			
PT-INR ≥ 1.6	4	(10.3)	9	(10.8)	1.000^b)^			
HbA1c ≥ 6.5 (NGSP %)	13	(34.2)	15	(22.4)	0.188^a)^			
HDL-cho < 40 (mg/dL)	9	(23.7)	15	(18.1)	0.472^a)^			
LDL-cho ≥ 120 (mg/dL)	7	(18.9)	21	(26.3)	0.387^a)^			
Triglyceride ≥ 150 (mg/dL)	6	(15.8)	10	(12.5)	0.626^a)^			
EF < 40 (%)	9	(23.1)	21	(23.1)	1.000^a)^			

† Logistic regression model including multivessel disease, history of surgery, history of bleeding, age, and anemia (*p* < 0.05 in univariate analysis)

* *p* < 0.05, (a) Chi–squared test, (b) Fisher’s exact test

OR, odds ratio = Exp (β); CI, confidence interval; BP, blood pressure; CABG, coronary artery bypass grafting; PCI, percutaneous coronary intervention; RCA, right coronary artery; LMT, left main trunk; LAD, left anterior descending coronary artery; LCX, left circumflex coronary artery; BMS, bare metal stent; DES, drug eluting stent; ACS, acute coronary syndrome; stable CAD, stable coronary artery diseases; AST, aspartate aminotransferase; ALT, alanine aminotransferase; eGFR, estimated glomerular filtration rate; CrCl, creatinine clearance; BNP, brain natriuretic peptide; PT-INR, prothrombin time-international normalized ratio; NGSP, national glycohemoglobin standardization program; HDL-C, high-density lipoprotein cholesterol; LDL-C, low-density lipoprotein cholesterol; EF, left ventricular ejection fraction

#### Subgroup analysis

The results of the subgroup analyses are presented in Additional file: Supplementary materials 6 and 7. In the population taking VKA at discharge, a history of bleeding was identified as a bleeding risk factor (OR: 5.000, 95% CI: 1.111–22.500, *p* = 0.036). In the population taking DOACs at discharge, history of surgery (OR: 4.785, 95% CI: 1.255–18.242, *p* = 0.022) and multivessel disease (OR: 2.929, 95% CI: 1.041–8.246, *p* = 0.042) were identified as bleeding risk factors.

#### Risk factors for major bleeding events

An AST level > 50 U/L was identified as an independent risk factor for major bleeding events (OR: 3.917, 95% CI: 1.333–11.506, *p* = 0.013) (Additional file: Supplementary material 8).

### Development and evaluation of the bleeding risk model

The predictive formula for the bleeding risk was created using age > 80 years, multivessel disease, history of surgery, low eGFR, anemia, and hypertension (Additional file: Supplementary material 4). There were no missing values for these factors. The integer scores determined using the ORs of these six factors were as follows: 1 point for eGFR 30–45 mL/min/1.73 m^2^; 1 point for age > 80 years; 1 point for multivessel disease; 1 point for hypertension; 1 point for anemia; 2 points for history of surgery; and 2 points for eGFR < 30 mL/min/1.73 m^2^ (Table 3).

**Table 3:** Development of a bleeding risk model in elderly patients with AF undergoing PCI

Variables	Category	Odds ratio	95% CI	Scores
eGFR ^a^ (mL/min/1.73m^2^)	< 30	1.534	0.798–2.951	+ 2
	30–45			+ 1
	> 45			+ 0
Age (years)	≤ 80 vs. > 80	2.023	0.834–4.905	+ 1
Multivessel disease	(–) vs. (+)	2.738	1.175–6.381	+ 1
History of surgery	(–) vs. (+)	3.661	1.311–10.221	+ 2
Hypertension	(–) vs. (+)	2.502	0.828–7.565	+ 1
Anemia ^b^	(–) vs. (+)	2.132	0.819–5.551	+ 1

Odds ratios = Exp(β); CI, confidence interval; eGFR, estimated glomerular filtration rate

a: eGFR was stratified into 3 groups (< 30, 30–45, > 45) (unit: mL/min/1.73m^2^)

b: Anemia was defined as a history of anemia or hemoglobin level < 11 (unit: g/dL)

**Fig. 1:**
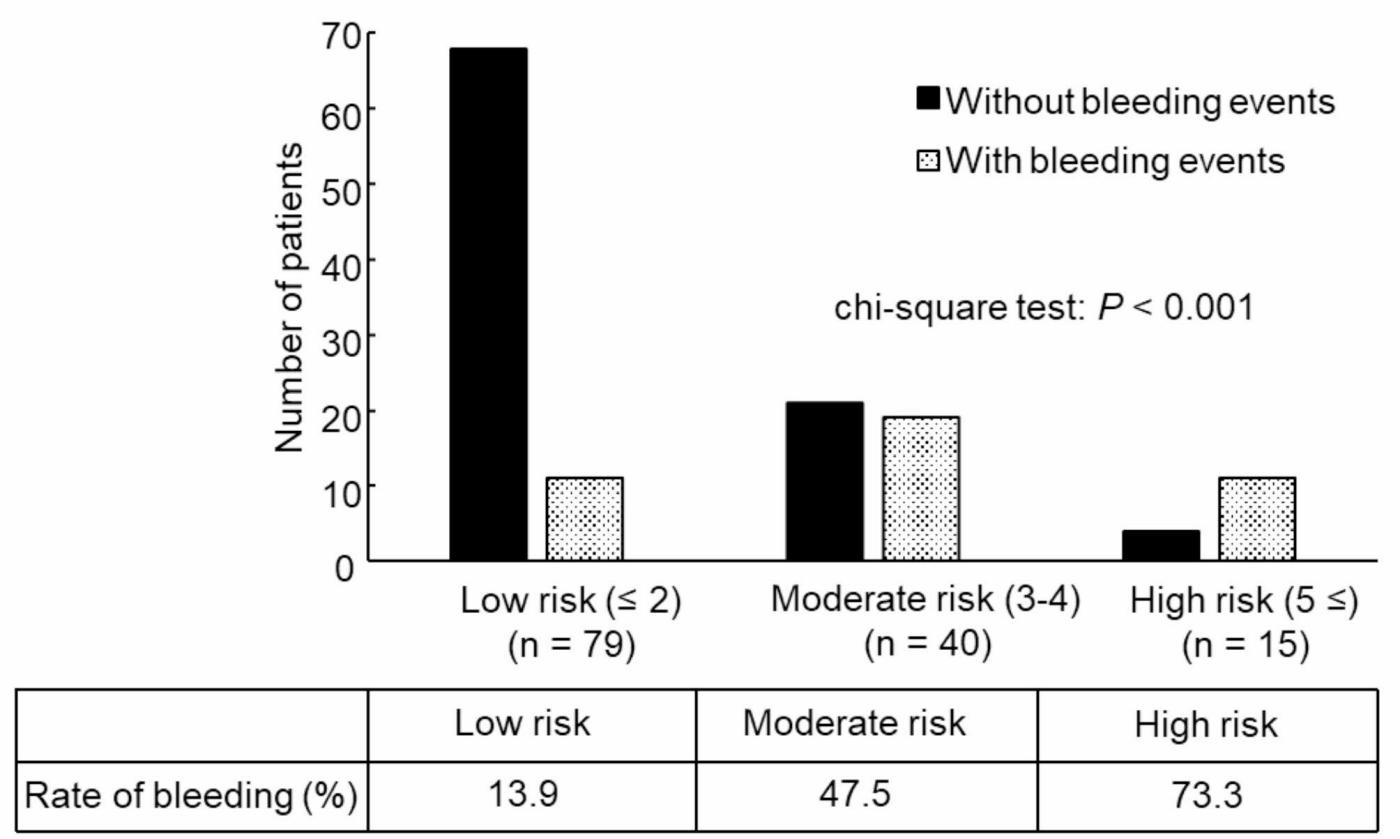
Evaluation of the bleeding risk model for elderly patients with AF undergoing primary PCI. Sensitivity: 0.27; specificity: 0.96; positive predictive value: 0.73; negative predictive value: 0.75; cut-off: 5 points

The sum of the scores of the six factors (range: 0–8 points) was calculated for all patients. A plot of the observed and predicted endpoints against the scores was obtained for 134 patients. The patients were stratified into three groups based on the calculated scores (Fig. 1). When the three groups were compared, those with scores ≥ 5 had a significantly higher rate of bleeding events than those with scores ≤ 2 (73.3% vs. 13.9%; *p* < 0.001); therefore, a score ≤ 2 was stratified as low risk, 3–4 as moderate risk, and ≥ 5 as high risk. For the high-risk group, the specificity was 96% and the positive predictive value was 73%. The C-statistic for bleeding events in the developed risk model was 0.75 (95% CI: 0.66–0.84; *p* < 0.001).

#### Association between antithrombotic use and bleeding events

**Fig. 2:**
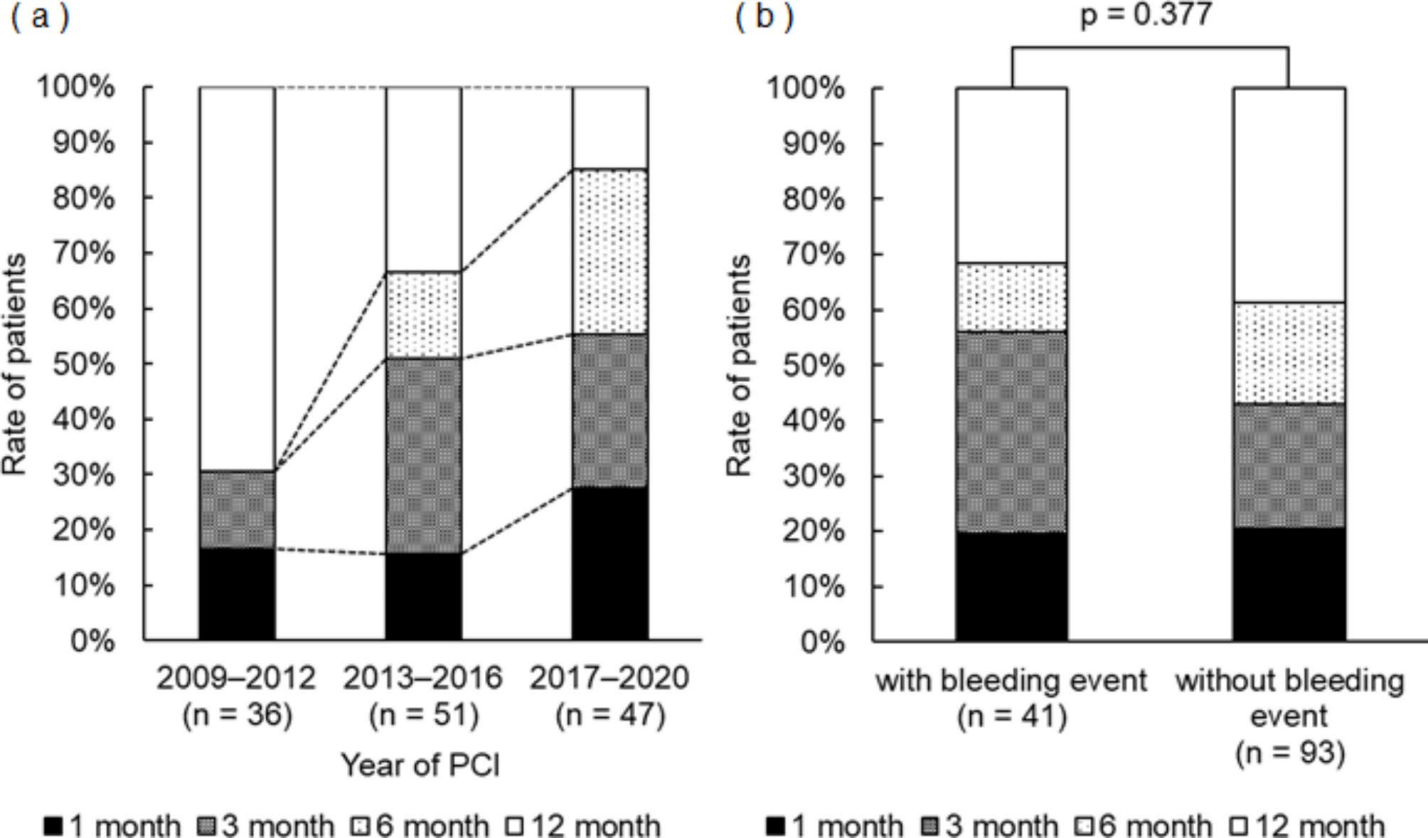
Relationship between year-to-year trends in concomitant use duration of triple anti-thrombotic therapy and bleeding events. **a**) Trends in duration of concomitant use of triple anti-thrombotic therapy by year of PCI. **b**) Relationship between duration of concomitant use of triple anti-thrombotic therapy and bleeding. Duration of concomitant use of triple antithrombotic therapy was collected at the end of follow-up. PCI, percutaneous coronary intervention

**Fig. 3:**
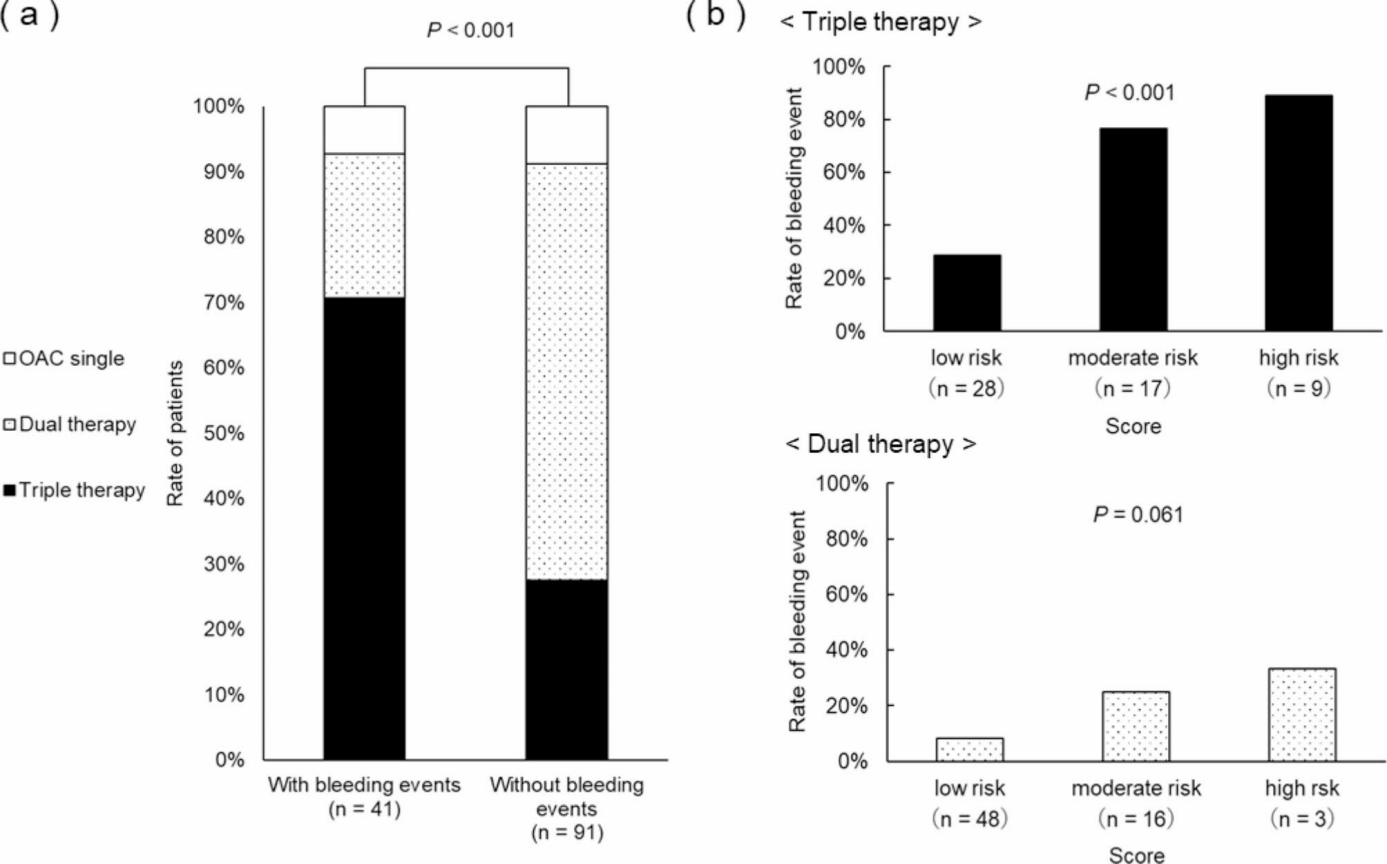
Relationship between number of antithrombotic agents and bleeding events. **a**) Relationship between number of antithrombotic agents and bleeding events. **b**) Bleeding rates by number of antithrombotic agents and between bleeding risk models. The number of antithrombotic agents was investigated at the time of bleeding in the bleeding event group and at the end of observation in the without-bleeding event group. OAC, oral anticoagulant

The year-by-year trend of DAPT duration is shown in Fig. 2a. It was observed that the duration of DAPT tended to become shorter over time, with fewer patients receiving DAPT for 12 months compared to the beginning of the study. The duration of DAPT was not significantly associated with bleeding events (Fig. 2b). The dose of DOACs at discharge was not significantly associated with bleeding events (Additional file: Supplementary material 9). The rate of triple therapy was significantly higher in the group with bleeding events than in the group without bleeding events (Fig. 3a). There was a significant difference in bleeding event rates between risk groups in the triple therapy group, whereas there was no significant difference in bleeding event rates between risk groups in the dual therapy group (Fig. 3b). Triple therapy at the time of bleeding or at the end of observation was identified as an independent risk factor for bleeding events (OR: 4.211, 95% CI: 1.978–8.965, p < 0.001) (Additional file: Supplementary material 10).

## Discussion

We found that age over 80 years, multivessel disease, and history of surgery were independent risk factors of bleeding events for elderly patients with AF who underwent PCI. To our knowledge, this is the first study to identify bleeding risk factors in an elderly population of patients with AF undergoing PCI. In addition, we found that the number of concomitant antithrombotic agents affects bleeding in elderly patients at high risk of bleeding. Bleeding events may be avoided by considering dual therapy after PCI in elderly patients at high risk of bleeding. Therefore, the results of this study may help to identify elderly patients at high risk of bleeding and assist in optimizing antithrombotic therapy to avoid bleeding events.

In the present study, patients older than 80 years exhibited a 2.5-fold higher risk of bleeding than those younger than 80 years. Older age was a consistent risk factor for bleeding events in previous studies [11]. The Japanese version of the high bleeding risk criteria also showed that the bleeding risk sharply increases at age 80 years and older [13]. Aging is associated with increased bleeding events because of decreased vascular elasticity, increased inflammatory cytokines, and increased comorbidities [17]. As populations age across the world, the number of very elderly patients with AF undergoing PCI is increasing [8]. However, many prospective clinical trials included only a small number of very elderly patients. As such, there is limited data on bleeding risk available in this population. Therefore, the results of our study have important clinical implications.

Further, we reported for the first time, to our knowledge, that multivessel disease is a risk factor for bleeding events in elderly patients with AF undergoing PCI. Patients with multivessel disease as a result of long-term hypertension and diabetes [18, 19], which are risk factors for atherosclerosis, have a high incidence of myocardial infarction [20]. Multivessel disease is a risk factor that reflects the effects of long-term complications of arteriosclerosis, which has been reported as a risk factor for epistaxis and gastrointestinal bleeding [21, 22]. Therefore, it is believed that the association between multivessel disease and bleeding events reflects an increased risk of bleeding events because of the long-term complications of arteriosclerosis.

In this study, 42% of patients with a history of surgery had undergone gastrointestinal surgery and 29% had undergone surgery related to malignancy. Potential gastrointestinal bleeding with coexisting gastrointestinal disease or bleeding tendency caused by cancer-related anemia may affect the bleeding events associated with anticoagulant therapy [23–25]. Additionally, because coagulation and fibrinolytic systems are activated after surgery, the administration of antithrombotic drugs promotes the bleeding tendency [26]. Therefore, it was thought that the risk of bleeding was increased due to factors such as gastrointestinal disease, concomitant malignancy, and the activation of coagulation and fibrinolytic systems after surgery.

AST > 50 U/L was identified as a risk factor for major bleeding, which differed from the risk factors for BARC class ≥ 2 bleeding. This discrepancy was attributed to the fact that approximately 60% of patients who experienced a bleeding event in this study fell into class 2 of the BARC criteria. While previous studies on bleeding risk factors primarily focused on major bleeding as the primary endpoint, this study recognized the importance of identifying bleeding risk factors, including BARC class 2 events. Such events have significant implications for patients’ quality of life and readmission rates [27]. The bleeding risk factors identified in this study could predict not only major bleeding but also BARC class 2 bleeding events requiring clinical attention.

In the Credo-Kyoto study [28], a large cohort of Japanese patients undergoing PCI was included, and 74.8% of the patients were male. However, in this current study, the proportion of male participants was slightly higher at 82.8% (111 patients). It has been reported that males with AF tend to be older [15]. Therefore, the higher proportion of males in this study compared to the previous report may be attributed to the age distribution of the participants.

The particularly high risk of bleeding in elderly patients with AF undergoing PCI is a clinical challenge. Therefore, optimizing antithrombotic therapy in elderly patients with AF undergoing PCI is important in clinical practice. The duration of triple antithrombotic therapy is being reduced to avoid the high bleeding risk associated with it [13]. On the other hand, some reports suggest that triple therapy after PCI is associated with an increased risk of bleeding, regardless of its duration [29]. A meta-analysis of large clinical trials involving patients with AF undergoing PCI revealed that dual therapy with an oral anticoagulant and P2Y12 inhibitor reduced bleeding events by 20–40% compared with triple therapy [30]. In this study, the rate of bleeding events was also higher with triple therapy than with dual therapy. In particular, a higher rate of bleeding events was seen in the high-risk group with triple therapy. Based on the above, in elderly patients at high risk of bleeding, dual therapy may be useful as an antithrombotic regimen after PCI. Our results may help to predict the risk of bleeding in elderly patients with AF undergoing PCI and to plan treatment according to risk.

The present study identified multivessel disease as a risk factor for bleeding in elderly patients with AF undergoing PCI. Although multivessel disease may reflect long-term complications of arteriosclerosis, the association between arteriosclerosis and bleeding has not been adequately investigated. Therefore, the association between findings of arteriosclerosis and bleeding in elderly patients with AF undergoing PCI should be investigated in the future. In addition, in elderly patients at high risk of bleeding, the efficacy and safety of dual therapy should be investigated in future multicenter prospective trials.

### Study limitations

This study had three main limitations. First, the follow-up period after PCI was 1 year, which might have resulted in the underestimation of actual long-term bleeding rates. However, the recommended duration for concomitant antiplatelet therapy for this patient population is a maximum of 1 year; therefore, the follow-up period was considered appropriate. Second, we did not collect information to directly assess atherosclerosis, such as intravascular ultrasound, cardio–ankle vascular index, ankle–brachial pressure index, or carotid ultrasound. These data may clarify why multivessel disease was identified as a risk factor for bleeding events. Similarly, information on polyvascular disease was not collected. Third, the developed risk model was not externally validated. The developed risk model will need to be validated in other populations in the future.

## Conclusions

Age over 80 years, multivessel disease, and history of surgery were risk factors of bleeding events for elderly patients with AF who underwent PCI. In addition, the number of concomitant antithrombotic agents was found to affect bleeding. Elderly patients at high risk of bleeding may avoid bleeding events by considering dual therapy after PCI. These results may help to predict bleeding risk in elderly patients with AF undergoing PCI and to plan treatment according to risk.

### Electronic supplementary material

Below is the link to the electronic supplementary material.

Additional File 1: Supplementary material 1 to 10.

## Data Availability

The data that support the findings of this study are available on reasonable request from the corresponding author. The data are not publicly available due to privacy or ethical restrictions.

## Abbreviations

AF: Atrial fibrillation
PCI: Percutaneous coronary intervention
HMG-CoA: Hydroxymethylglutaryl-coenzyme A
OAC: Oral anticoagulant
DAPT: Dual antiplatelet therapy
eGFR: Estimated glomerular filtration rate
HbA1c: Glycosylated hemoglobin
BARC: Bleeding academic research consortium
DOACs: Direct oral anticoagulants
CrCl: Creatinine clearance
OR: Odds ratio; CI: Confidence interval
