# Supplementary material for: Bleeding risk factors and real-world antithrombotic therapies in elderly patients with atrial fibrillation undergoing percutaneous coronary intervention: a retrospective study
Source: J Pharm Health Care Sci. 2023 Dec 12;9:41. doi: 10.1186/s40780-023-00308-8 (PMC10714450; doi:10.1186/s40780-023-00308-8)
Supplement: Supplementary file 1 — Additional File 1: Supplementary material 1 to 10. [file 40780_2023_308_MOESM1_ESM.docx]

**Supplementary Material**

*Journal of Pharmaceutical Health Care and Sciences*

**Bleeding risk factors and real-world antithrombotic therapies in elderly patients with atrial fibrillation undergoing percutaneous coronary intervention: A retrospective study**

Kanako Fujita (BPharm)^1,2,*^, Noriko Kohyama (PhD)^1^, Miki Sato (PhD)^1^, Tomokazu Deguchi (PhD)^1,2^, Hiroshi Suzuki (MD, PhD)^3^, Mio Ebato (MD, PhD)^3^, Mari Kogo (PhD)^1^

^1^ Division of Pharmacotherapeutics, Department of Clinical Pharmacy, Showa University School of Pharmacy, Tokyo, Japan

^2^ Department of Pharmacy, Showa University Fujigaoka Hospital, Yokohama, Japan

^3^ Division of Cardiology, Department of Internal Medicine, Showa University Fujigaoka Hospital, Yokohama, Japan

^∗^ **Corresponding author:** Kanako Fujita (BPharm); E-mail: k.fujita0904@pharm.showa-u.ac.jp

Patients undergoing

primary PCI

(n=2608)

Patients undergoing

primary PCI with AF

(Age

≥

65)

(n=201)

Analysis object

(n=134)

67 excluded

28 not taking anticoagulants

8 scheduled surgery

8 valve replacement

4 aneurysm complication

14 lost to follow up

5

events related to the PCI

With bleeding events

(n=41)

Without bleeding events

(n=93)

**Supplementary material 1** Study flow chart

**Supplementary material 2** Investigation of cut-off values for various age groups

| Cut-off value (years) | C-statistic | 95% CI | Sensitivity | Specificity |
| --- | --- | --- | --- | --- |
| > 70 | 0.502 | 0.395–0.608 | 0.756 | 0.247 |
| > 75 | 0.531 | 0.425–0.637 | 0.601 | 0.452 |
| > 80 | 0.594 | 0.487–0.702 | 0.415 | 0.774 |
| > 85 | 0.542 | 0.434–0.651 | 0.176 | 0.914 |
| > 90 | 0.495 | 0.389–0.601 | 0.000 | 0.989 |

CI, confidence interval

**Supplementary material 3** Comparison of C-statistic for various bleeding risk factors

| Risk factor | C-statistic | *p*-value | 95% CI |
| --- | --- | --- | --- |
| Anemia | 0.585 | 0.12 | (0.48–0.69) |
| Smoking history | 0.581 | 0.14 | (0.48–0.68) |
| eGFR (mL/min/1.73m^2^)^a)^ | 0.574 | 0.18 | (0.46–0.68) |
| CrCl (mL/min)^b)^ | 0.449 | 0.35 | (0.34–0.56) |
| History of bleeding | 0.574 | 0.18 | (0.46–0.68) |
| Hypertension | 0.572 | 0.19 | (0.47–0.67) |
| Malignancy | 0.541 | 0.45 | (0.43–0.65) |
| Heart failure | 0.538 | 0.48 | (0.43–0.65) |
| Liver disease | 0.510 | 0.86 | (0.40–0.62) |
| Cerebral infarction | 0.489 | 0.83 | (0.38–0.59) |

a) eGFR was stratified into 3 groups (< 30, 30–45, > 45) (unit: mL/min/1.73m^2^).

b) CrCl was stratified into 3 groups (< 30, 30–50, > 50) (unit: mL/min).

CI, confidence interval; eGFR, estimated glomerular filtration rate; CrCl, creatinine clearance

**Supplementary material 4** Comparison of the C-statistic for the bleeding risk model formula

|  | Odds ratio（95% CI) | | | | | | | |
| --- | --- | --- | --- | --- | --- | --- | --- | --- |
| Risk factor | model 1 | | model 2 | | model 3 | | model 4 | |
| Multivessel disease | 2.74 | (1.18–6.38) | 2.82 | (1.22–6.54) | 2.49 | (1.06–5.88) | 2.48 | (1.08–5.73) |
| Age (> 80 years) | 2.02 | (0.83–4.91) | 2.08 | (0.85–5.08) | 2.21 | (0.92–5.32) | 2.21 | (0.93–5.29) |
| History of surgery | 3.66 | (1.31–10.22) | 3.52 | (1.25–9.89) | 3.21 | (1.17–8.83) | 3.18 | (1.17–8.63) |
| Anemia | 2.13 | (0.82–5.55) | 2.33 | (0.90–6.06) | 1.98 | (0.76–5.18) | 1.98 | (0.77–5.09) |
| Smoking history |  |  |  |  |  |  | 1.58 | (0.66–3.80) |
| eGFR (mL/min/m^2^)^a)^ | 1.53 | (0.80–2.95) |  |  | 1.70 | (0.90–3.23) | 1.69 | (0.89–3.23) |
| CrCl (mL/min)^b)^ |  |  | 0.98 | (0.53–1.81) |  |  |  |  |
| History of bleeding |  |  |  |  | 1.21 | (0.38–3.90) |  |  |
| Hypertension | 2.50 | (0.83–7.57) | 2.81 | (0.91–8.65) |  |  |  |  |
| C-statistic (95% CI) | 0.76 (0.67–0.85) | | 0.74 (0.65–0.84) | | 0.75 (0.66–0.85) | | 0.75 (0.66–0.84) | |

a) eGFR was stratified into 3 groups (< 30, 30–45, > 45) (unit: mL/min/1.73m^2^).

b) CrCl was stratified into 3 groups (< 30, 30–50, > 50) (unit: mL/min).

CI, confidence interval; eGFR, estimated glomerular filtration rate; CrCl, creatinine clearance

25 (

61.0

%)

10 (

24.4

%)

3 (

7.3

%)

1 (2.4%)

2 (

4.9

%)

Type 2

Type 3a

Type 3b

Type 3c

Type 5

a

4 (

9.8

%)

9 (

22.0

%)

15 (

36.6

%)

3 (

7.3

%)

1(

2.4

%)

9 (

22.0

%)

Subcutaneous bleed

Nose bleed

Gastrointestinal bleed

Hemorrhoids

Intracranial or retroperitoneal bleed

Others

b

**Supplementary material 5** Bleeding events that occurred in elderly patients with AF within 1 year after PCI

(a): Incidence of bleeding according to BARC criteria. (b): Details of bleeding site.

**Supplementary material 6** Bleeding risk factors in elderly patients with AF undergoing PCI in the population receiving VKA at discharge

| Variables | With bleeding events (n = 14) | | Without bleeding events (n = 40) | | Univariate analysis |  | Multivariate analysis^†^ (stepwise selection method) | |
| --- | --- | --- | --- | --- | --- | --- | --- | --- |
|  |  |  |  |  | *p*-value |  | OR (95% CI) | *p*-value |
|  | n (%) | | n (%) | |  |  |  |  |
| Age > 80 years | 5 | (35.7) | 7 | (17.5) | 0.261^a)^ |  |  |  |
| Sex, male | 11 | (78.6) | 38 | (95.0) | 0.103^a)^ |  |  |  |
| Body mass index < 20 (kg/m^2^) | 4 | (28.6) | 8 | (20.0) | 0.485^a)^ |  |  |  |
| Smoking history | 9 | (64.3) | 24 | (60.0) | 0.777^b)^ |  |  |  |
| Alcohol consumption history | 5 | (35.7) | 24 | (60.0) | 0.117^b)^ |  |  |  |
| Systolic BP ≥ 140 (mmHg) | 2 | (14.3) | 8 | (20.0) | 1.000^a)^ |  |  |  |
| Diastolic BP ≥ 90 (mmHg) | 1 | (7.1) | 1 | (2.5) | 0.455^a)^ |  |  |  |
| History of surgery | 3 | (21.4) | 6 | (15.0) | 0.681^a)^ |  |  |  |
| History of bleeding | 5 | (35.7) | 4 | (10.0) | 0.041^a)*^ |  | 5.000 (1.111–22.500) | 0.036^*^ |
| History of CABG | 0 | (0.0) | 2 | (5.0) | 1.000^a)^ |  |  |  |
| Concomitant disease  or medical history |  |  |  |  |  |  |  |  |
| Hypertension | 13 | (92.9) | 26 | (65.0) | 0.080^a)^ |  |  |  |
| Dyslipidemia | 7 | (50.0) | 18 | (45.0) | 0.747^b)^ |  |  |  |
| Diabetes mellitus | 7 | (50.0) | 11 | (27.5) | 0.188^a)^ |  |  |  |
| Cerebral infarction | 2 | (14.3) | 6 | (15.0) | 1.000^a)^ |  |  |  |
| Myocardial infection | 3 | (21.4) | 4 | (10.0) | 0.358^b)^ |  |  |  |
| Heart failure | 9 | (64.3) | 14 | (35.0) | 0.056^b)^ |  |  |  |
| Malignancy | 4 | (28.6) | 8 | (20.0) | 0.485^a)^ |  |  |  |
| Dialysis | 2 | (14.3) | 3 | (7.5) | 0.595^a)^ |  |  |  |
| Liver disease | 0 | (0.0) | 3 | (7.5) | 0.560^a)^ |  |  |  |
| Anemia | 7 | (50.0) | 9 | (22.5) | 0.087^a)^ |  |  |  |
| Gastrointestinal disease | 7 | (50.0) | 10 | (25.0) | 0.103^a)^ |  |  |  |
| Peripheral arterial disease | 4 | (28.6) | 6 | (15.0) | 0.424^b)^ |  |  |  |
| PCI profile |  |  |  |  |  |  |  |  |
| Number of target lesions ≥ 2 | 6 | (42.9) | 16 | (40.0) | 0.851^b)^ |  |  |  |
| Lesion site |  |  |  |  | 0.877^b)^ |  |  |  |
| RCA | 3 | (21.4) | 6 | (15.0) |  |  |  |  |
| LMT | 1 | (7.1) | 3 | (7.5) |  |  |  |  |
| LAD | 7 | (50.0) | 25 | (62.5) |  |  |  |  |
| LCX | 3 | (21.4) | 6 | (15.0) |  |  |  |  |
| Chronic total occlusion | 3 | (21.4) | 8 | (20.0) | 1.000^a)^ |  |  |  |
| Multivessel disease | 9 | (64.3) | 20 | (50.0) | 0.356^b)^ |  |  |  |
| Stent type |  |  |  |  | 0.328^a)^ |  |  |  |
| BMS | 6 | (42.9) | 11 | (27.5) |  |  |  |  |
| DES | 8 | (57.1) | 29 | (72.5) |  |  |  |  |
| Clinical presentation |  |  |  |  | 0.147^b)^ |  |  |  |
| ACS | 8 | (57.1) | 14 | (35.0) |  |  |  |  |
| stable CAD | 6 | (42.9) | 26 | (65.0) |  |  |  |  |
| Laboratory data |  |  |  |  |  |  |  |  |
| Albumin < 3.5 (g/dL) | 7 | (50.0) | 14 | (35.0) | 0.322^b)^ |  |  |  |
| White blood cell ≥ 10000 (/μL) | 4 | (28.6) | 3 | (7.5) | 0.065^a)^ |  |  |  |
| Hemoglobin < 11 (g/dL) | 5 | (35.7) | 8 | (20.0) | 0.285^a)^ |  |  |  |
| Platelet < 10 (10^4^/μL) | 1 | (7.1) | 0 | (0.0) | 0.259^a)^ |  |  |  |
| AST > 50 (U/L) | 6 | (42.9) | 8 | (20.0) | 0.154^a)^ |  |  |  |
| ALT > 50 (U/L) | 2 | (14.3) | 5 | (12.5) | 1.000^a)^ |  |  |  |
| eGFR (mL/min/1.73m^2^) |  |  |  |  | 0.066^a)^ |  |  |  |
| < 30 | 4 | (28.6) | 4 | (10.0) |  |  |  |  |
| 30–45 | 3 | (21.4) | 4 | (10.0) |  |  |  |  |
| > 45 | 7 | (50.0) | 32 | (80.0) |  |  |  |  |
| CrCl (mL/min) |  |  |  |  | 0.188^a)^ |  |  |  |
| < 30 | 2 | (14.3) | 4 | (10.0) |  |  |  |  |
| 30–50 | 7 | (50.0) | 11 | (27.5) |  |  |  |  |
| > 50 | 5 | (35.7) | 25 | (62.5) |  |  |  |  |
| Creatinine kinase > 230 (IU/L) | 5 | (35.7) | 11 | (27.5) | 0.735^a)^ |  |  |  |
| C-reactive protein > 0.3 (mg/L) | 9 | (64.3) | 18 | (51.4) | 0.414^b)^ |  |  |  |
| BNP ≥ 100 (pg/mL) | 11 | (91.7) | 28 | (80.0) | 0.659^a)^ |  |  |  |
| PT-INR ≥ 1.6 | 3 | (23.1) | 7 | (18.4) | 0.701^a)^ |  |  |  |
| HbA1c ≥ 6.5 (NGSP %) | 4 | (30.8) | 3 | (10.7) | 0.181^a)^ |  |  |  |
| HDL-cho < 40 (mg/dL) | 3 | (21.4) | 8 | (21.6) | 1.000^a)^ |  |  |  |
| LDL-cho ≥ 120 (mg/dL) | 2 | (15.4) | 7 | (20.0) | 1.000^a)^ |  |  |  |
| Triglyceride ≥ 150 (mg/dL) | 1 | (7.1) | 2 | (5.7) | 1.000^a)^ |  |  |  |
| EF < 40 (%) | 3 | (23.1) | 10 | (26.3) | 1.000^a)^ |  |  |  |
| † Logistic regression model including history of bleeding (*p* < 0.05 in univariate analysis).  * *p* < 0.05, a) Fisher’s exact test, b) Chi–squared test  OR, odds ratio = Exp (β); CI, confidence interval; BP, blood pressure; CABG, coronary artery bypass grafting; PCI, percutaneous coronary intervention; RCA, right coronary artery; LMT, left main trunk; LAD, left anterior descending coronary artery; LCX, left circumflex coronary artery; BMS, bare metal stent; DES, drug eluting stent; ACS, acute coronary syndrome; stable CAD, stable coronary artery diseases; AST, aspartate aminotransferase; ALT, alanine aminotransferase; eGFR, estimated glomerular filtration rate; CrCl, creatinine clearance; BNP, brain natriuretic peptide; PT-INR, prothrombin time-international normalized ratio; NGSP, national glycohemoglobin standardization program; HDL-C, high-density lipoprotein cholesterol; LDL-C, low-density lipoprotein cholesterol; EF, left ventricular ejection fraction. | | | | | | | | |

**Supplementary material 7** Bleeding risk factors in elderly patients with AF undergoing PCI　in the population receiving DOACs at discharge

| Variables | With bleeding events (n = 27) | | Without bleeding events (n = 53) | | Univariate analysis |  | Multivariate analysis^†^ (stepwise selection method) | |
| --- | --- | --- | --- | --- | --- | --- | --- | --- |
|  |  |  |  |  | *p*-value |  | OR (95% CI) | *p*-value |
|  | n (%) | | n (%) | |  |  |  |  |
| Age > 80 years | 12 | (44.4) | 14 | (26.4) | 0.104^a)^ |  |  |  |
| Sex, male | 21 | (77.8) | 41 | (77.4) | 0.966^a)^ |  |  |  |
| Body mass index < 20 (kg/m^2^) | 7 | (25.9) | 7 | (13.2) | 0.214^b)^ |  |  |  |
| Smoking history | 21 | (77.8) | 29 | (54.7) | 0.044^a)*^ |  |  |  |
| Alcohol consumption history | 13 | (50.0) | 20 | (37.7) | 0.299^a)^ |  |  |  |
| Systolic BP ≥ 140 (mmHg) | 4 | (14.8) | 13 | (24.5) | 0.315^a)^ |  |  |  |
| Diastolic BP ≥ 90 (mmHg) | 3 | (11.1) | 6 | (11.3) | 1.000^b)^ |  |  |  |
| History of surgery | 9 | (33.3) | 4 | (7.5) | 0.008^b)*^ |  | 4.785 (1.255–18.242) | 0.022^*^ |
| History of bleeding | 5 | (18.5) | 5 | (9.4) | 0.293^b)^ |  |  |  |
| History of CABG | 3 | (11.1) | 1 | (1.9) | 0.106^b)^ |  |  |  |
| Concomitant disease  or medical history |  |  |  |  |  |  |  |  |
| Hypertension | 22 | (81.5) | 40 | (75.5) | 0.543^a)^ |  |  |  |
| Dyslipidemia | 19 | (70.4) | 27 | (50.9) | 0.096^a)^ |  |  |  |
| Diabetes mellitus | 8 | (29.6) | 20 | (37.7) | 0.472^a)^ |  |  |  |
| Cerebral infarction | 5 | (18.5) | 12 | (22.6) | 0.670^a)^ |  |  |  |
| Myocardial infection | 2 | (7.4) | 7 | (13.2) | 0.710^b)^ |  |  |  |
| Heart failure | 10 | (37.0) | 22 | (41.5) | 0.699^a)^ |  |  |  |
| Malignancy | 6 | (22.2) | 7 | (13.2) | 0.345^a)^ |  |  |  |
| Dialysis | 0 | (0.0) | 0 | (0.0) | - |  |  |  |
| Liver disease | 3 | (11.1) | 2 | (3.8) | 0.329^b)^ |  |  |  |
| Anemia | 7 | (25.9) | 7 | (13.2) | 0.214^a)^ |  |  |  |
| Gastrointestinal disease | 6 | (22.2) | 7 | (13.2) | 0.345^b)^ |  |  |  |
| Peripheral arterial disease | 1 | (3.7) | 5 | (9.4) | 0.658^b)^ |  |  |  |
| PCI profile |  |  |  |  |  |  |  |  |
| Number of target lesions ≥ 2 | 9 | (33.3) | 17 | (32.1) | 0.910^a)^ |  |  |  |
| Lesion site |  |  |  |  | 0.933^b)^ |  |  |  |
| RCA | 7 | (25.9) | 15 | (28.3) |  |  |  |  |
| LMT | 0 | (0.0) | 1 | (1.9) |  |  |  |  |
| LAD | 15 | (55.6) | 29 | (54.7) |  |  |  |  |
| LCX | 5 | (18.5) | 8 | (15.1) |  |  |  |  |
| Chronic total occlusion | 8 | (29.6) | 14 | (26.4) | 0.761^a)^ |  |  |  |
| Multivessel disease | 19 | (70.4) | 21 | (39.6) | 0.009^a)*^ |  | 2.929 (1.041–8.246) | 0.042^*^ |
| Stent type |  |  |  |  | 0.683^b)^ |  |  |  |
| BMS | 3 | (11.1) | 4 | (7.5) |  |  |  |  |
| DES | 24 | (88.9) | 49 | (92.5) |  |  |  |  |
| Clinical presentation |  |  |  |  | 1.000^a)^ |  |  |  |
| ACS | 14 | (51.9) | 27 | (50.9) |  |  |  |  |
| stable CAD | 13 | (48.1) | 26 | (49.1) |  |  |  |  |
| Laboratory data |  |  |  |  |  |  |  |  |
| Albumin < 3.5 (g/dL) | 7 | (25.9) | 15 | (28.3) | 0.822^a)^ |  |  |  |
| White blood cell ≥ 10000 (/μL) | 5 | (18.5) | 7 | (13.2) | 0.527^b)^ |  |  |  |
| Hemoglobin < 11 (g/dL) | 4 | (14.8) | 7 | (13.2) | 1.000^b)^ |  |  |  |
| Platelet < 10 (10^4^/μL) | 1 | (3.7) | 1 | (1.9) | 1.000^b)^ |  |  |  |
| AST > 50 (U/L) | 7 | (25.9) | 11 | (20.8) | 0.600^a)^ |  |  |  |
| ALT > 50 (U/L) | 4 | (14.8) | 8 | (15.1) | 1.000^b)^ |  |  |  |
| eGFR (mL/min/1.73m^2^) |  |  |  |  | 0.184^b)^ |  |  |  |
| < 30 | 2 | (7.4) | 0 | (0.0) |  |  |  |  |
| 30–45 | 4 | (14.8) | 9 | (17.0) |  |  |  |  |
| > 45 | 21 | (77.8) | 44 | (83.0) |  |  |  |  |
| CrCl (mL/min) |  |  |  |  | 0.024^a)*^ |  |  |  |
| < 30 | 6 | (22.2) | 3 | (5.7) |  |  |  |  |
| 30–50 | 6 | (22.2) | 25 | (47.2) |  |  |  |  |
| > 50 | 15 | (55.6) | 25 | (47.2) |  |  |  |  |
| Creatinine kinase > 230 (IU/L) | 3 | (11.5) | 12 | (22.6) | 0.362^b)^ |  |  |  |
| C-reactive protein > 0.3 (mg/L) | 12 | (52.2) | 22 | (48.9) | 0.798^a)^ |  |  |  |
| BNP ≥ 100 (pg/mL) | 17 | (65.4) | 37 | (71.2) | 0.603^a)^ |  |  |  |
| PT-INR ≥ 1.6 | 1 | (3.8) | 3 | (6.7) | 1.000^b)^ |  |  |  |
| HbA1c ≥ 6.5 (NGSP %) | 10 | (40.0) | 14 | (35.9) | 0.741^a)^ |  |  |  |
| HDL-cho < 40 (mg/dL) | 6 | (25.0) | 7 | (15.2) | 0.346^a)^ |  |  |  |
| LDL-cho ≥ 120 (mg/dL) | 5 | (20.8) | 14 | (31.1) | 0.363^a)^ |  |  |  |
| Triglyceride ≥ 150 (mg/dL) | 5 | (20.8) | 8 | (17.8) | 0.756^b)^ |  |  |  |
| EF < 40 (%) | 6 | (23.1) | 11 | (20.8) | 0.813^a)^ |  |  |  |
| † Logistic regression model including smoking history, history of surgery, multivessel disease, and CrCl (< 30, 30–50, 50 <) (*p* < 0.05 in univariate analysis).  * *p* < 0.05, a) Chi–squared test, b) Fisher’s exact test  OR, odds ratio = Exp (β); CI, confidence interval; BP, blood pressure; CABG, coronary artery bypass grafting; PCI, percutaneous coronary intervention; RCA, right coronary artery; LMT, left main trunk; LAD, left anterior descending coronary artery; LCX, left circumflex coronary artery; BMS, bare metal stent; DES, drug eluting stent; ACS, acute coronary syndrome; stable CAD, stable coronary artery diseases; AST, aspartate aminotransferase; ALT, alanine aminotransferase; eGFR, estimated glomerular filtration rate; CrCl, creatinine clearance; BNP, brain natriuretic peptide; PT-INR, prothrombin time-international normalized ratio; NGSP, national glycohemoglobin standardization program; HDL-C, high-density lipoprotein cholesterol; LDL-C, low-density lipoprotein cholesterol; EF, left ventricular ejection fraction. | | | | | | | | |

**Supplementary material 8** Major bleeding risk factors in elderly patients with AF undergoing PCI

| Variables | With bleeding events (n = 16) | | Without bleeding events (n = 118) | | Univariate analysis |  | Multivariate analysis^†^ (stepwise selection method) | |
| --- | --- | --- | --- | --- | --- | --- | --- | --- |
|  |  |  |  |  | *p*-value |  | OR (95% CI) | *p*-value |
|  | n (%) | | n (%) | |  |  |  |  |
| Age > 80 years | 6 | (37.5) | 32 | (27.1) | 0.388^a)^ |  |  |  |
| Sex, male | 14 | (87.5) | 97 | (82.2) | 1.000^a)^ |  |  |  |
| Body mass index < 20 (kg/m^2^) | 4 | (25.0) | 22 | (18.6) | 0.513^a)^ |  |  |  |
| Smoking history | 12 | (75.0) | 71 | (60.2) | 0.252^b)^ |  |  |  |
| Alcohol consumption history | 6 | (37.5) | 56 | (47.9) | 0.436^b)^ |  |  |  |
| Systolic BP ≥ 140 (mmHg) | 2 | (12.5) | 25 | (21.2) | 0.525^a)^ |  |  |  |
| Diastolic BP ≥ 90 (mmHg) | 1 | (6.3) | 10 | (8.5) | 1.000^a)^ |  |  |  |
| History of surgery | 3 | (18.8) | 19 | (16.1) | 0.727^a)^ |  |  |  |
| History of bleeding | 3 | (18.8) | 16 | (13.6) | 0.701^a)^ |  |  |  |
| History of CABG | 0 | (0.0) | 6 | (5.1) | 1.000^a)^ |  |  |  |
| Concomitant disease  or medical history |  |  |  |  |  |  |  |  |
| Hypertension | 15 | (93.8) | 86 | (72.9) | 0.118^a)^ |  |  |  |
| Dyslipidemia | 6 | (37.5) | 65 | (55.1) | 0.186^b)^ |  |  |  |
| Diabetes mellitus | 5 | (31.3) | 41 | (34.7) | 0.782^b)^ |  |  |  |
| Cerebral infarction | 4 | (25.0) | 21 | (17.8) | 0.499^a)^ |  |  |  |
| Myocardial infection | 1 | (6.3) | 15 | (12.7) | 0.692^a)^ |  |  |  |
| Heart failure | 6 | (37.5) | 49 | (41.5) | 0.759^b)^ |  |  |  |
| Malignancy | 4 | (25.0) | 21 | (17.8) | 0.499^a)^ |  |  |  |
| Dialysis | 2 | (12.5) | 3 | (2.5) | 0.108^b)^ |  |  |  |
| Liver disease | 0 | (0.0) | 8 | (6.8) | 0.595^a)^ |  |  |  |
| Anemia | 6 | (37.5) | 24 | (20.3) | 0.196^a)^ |  |  |  |
| Gastrointestinal disease | 5 | (31.3) | 25 | (21.2) | 0.353^a)^ |  |  |  |
| Peripheral arterial disease | 2 | (12.5) | 14 | (11.9) | 1.000^a)^ |  |  |  |
| PCI profile |  |  |  |  |  |  |  |  |
| Number of target lesions ≥ 2 | 6 | (37.5) | 42 | (35.6) | 0.881^b)^ |  |  |  |
| Lesion site |  |  |  |  | 0.721^a)^ |  |  |  |
| RCA | 4 | (25.0) | 27 | (22.9) |  |  |  |  |
| LMT | 1 | (6.3) | 4 | (3.4) |  |  |  |  |
| LAD | 8 | (50.0) | 68 | (57.6) |  |  |  |  |
| LCX | 3 | (18.8) | 19 | (16.1) |  |  |  |  |
| Chronic total occlusion | 5 | (31.3) | 28 | (23.7) | 0.541^a)^ |  |  |  |
| Multivessel disease | 11 | (68.8) | 58 | (49.2) | 0.141^b)^ |  |  |  |
| Stent type |  |  |  |  | 0.164^a)^ |  |  |  |
| BMS | 5 | (31.3) | 19 | (16.1) |  |  |  |  |
| DES | 11 | (68.8) | 99 | (83.9) |  |  |  |  |
| Clinical presentation |  |  |  |  | 0.186^b)^ |  |  |  |
| ACS | 10 | (62.5) | 53 | (44.9) |  |  |  |  |
| stable CAD | 6 | (37.5) | 65 | (55.1) |  |  |  |  |
| Laboratory data |  |  |  |  |  |  |  |  |
| Albumin < 3.5 (g/dL) | 6 | (37.5) | 37 | (31.4) | 0.621^b)^ |  |  |  |
| White blood cell ≥ 10000 (/μL) | 4 | (25.0) | 15 | (12.7) | 0.244^a)^ |  |  |  |
| Hemoglobin < 11 (g/dL) | 3 | (18.8) | 21 | (17.8) | 1.000^a)^ |  |  |  |
| Platelet < 10 (10^4^/μL) | 1 | (6.3) | 2 | (1.7) | 0.319^b)^ |  |  |  |
| AST > 50 (U/L) | 8 | (50.0) | 24 | (20.3) | 0.024^a)*^ |  | 3.917 (1.333–11.506) | 0.013^*^ |
| ALT > 50 (U/L) | 3 | (18.8) | 16 | (13.6) | 0.701^a)^ |  |  |  |
| eGFR (mL/min/1.73m^2^) |  |  |  |  | 0.021^a)*^ |  |  |  |
| < 30 | 4 | (25.0) | 6 | (5.1) |  |  |  |  |
| 30–45 | 1 | (6.3) | 19 | (16.1) |  |  |  |  |
| > 45 | 11 | (68.8) | 93 | (78.8) |  |  |  |  |
| CrCl (mL/min) |  |  |  |  | 0.093^a)^ |  |  |  |
| < 30 | 3 | (18.8) | 12 | (10.2) |  |  |  |  |
| 30–50 | 2 | (12.5) | 47 | (39.8) |  |  |  |  |
| > 50 | 11 | (68.8) | 59 | (50.0) |  |  |  |  |
| Creatinine kinase > 230 (IU/L) | 6 | (37.5) | 25 | (21.4) | 0.204^a)^ |  |  |  |
| C-reactive protein > 0.3 (mg/L) | 9 | (56.3) | 52 | (51.5) | 0.723^b)^ |  |  |  |
| BNP ≥ 100 (pg/mL) | 12 | (85.7) | 81 | (73.0) | 0.516^a)^ |  |  |  |
| PT-INR ≥ 1.6 | 1 | (6.7) | 13 | (12.1) | 1.000^a)^ |  |  |  |
| HbA1c ≥ 6.5 (NGSP %) | 4 | (26.7) | 27 | (30.0) | 1.000^a)^ |  |  |  |
| HDL-cho < 40 (mg/dL) | 5 | (31.3) | 19 | (18.1) | 0.309^a)^ |  |  |  |
| LDL-cho ≥ 120 (mg/dL) | 2 | (12.5) | 26 | (25.7) | 0.351^a)^ |  |  |  |
| Triglyceride ≥ 150 (mg/dL) | 3 | (18.8) | 13 | (12.7) | 0.454^a)^ |  |  |  |
| EF < 40 (%) | 3 | (20.0) | 27 | (23.5) | 1.000^a)^ |  |  |  |
| † Logistic regression model including AST > 50, eGFR (< 30, 30–45, 45 <) (*p* < 0.05 in univariate analysis).  * *p* < 0.05, a) Fisher’s exact test, b) Chi–squared test  OR, odds ratio = Exp (β); CI, confidence interval; BP, blood pressure; CABG, coronary artery bypass grafting; PCI, percutaneous coronary intervention; RCA, right coronary artery; LMT, left main trunk; LAD, left anterior descending coronary artery; LCX, left circumflex coronary artery; BMS, bare metal stent; DES, drug eluting stent; ACS, acute coronary syndrome; stable CAD, stable coronary artery diseases; AST, aspartate aminotransferase; ALT, alanine aminotransferase; eGFR, estimated glomerular filtration rate; CrCl, creatinine clearance; BNP, brain natriuretic peptide; PT-INR, prothrombin time-international normalized ratio; NGSP, national glycohemoglobin standardization program; HDL-C, high-density lipoprotein cholesterol; LDL-C, low-density lipoprotein cholesterol; EF, left ventricular ejection fraction. | | | | | | | | |

5

(18.5%)

14

(27.0%)

14 (51.9%)

16 (30.8%)

6

(22.2%)

12

(23.1%)

2

(7.4%)

10

(19.2%)

0%

10%

20%

30%

40%

50%

60%

70%

80%

90%

100%

with bleeding

（

n = 27

）

without bleeding

（

n = 52

）

Number (%)

standard dose

low dose

under dose

over dose

*p*

= 0.

236

**Supplementary material 9** Relationship between DOAC dose and bleeding events

**Supplementary material 10** Relationship between the number of antithrombotic agents and bleeding events

| Variables | Multivariate analysis^a)^ (stepwise selection method) | |
| --- | --- | --- |
|  | OR (95% CI) | *p*-value |
| Age > 80 years | 2.657 (1.064–6.636) | 0.036 |
| History of surgery | 3.738 (1.306–10.697) | 0.014 |
| Multivessel disease | 3.165 (1.302–7.693) | 0.011 |
| Number of antithrombotic agents^b)^ | 4.211 (1.978–8.965) | < 0.001 |

a) Logistic regression model including multivessel disease, history of surgery, history of bleeding, age, anemia, and number of antithrombotic agents (*p* < 0.05 in univariate analysis).

b) Categorized into 3 groups: triple therapy, dual therapy, and OAC single. Number of antithrombotic agents was investigated at the time of bleeding in the bleeding event group and the end of observation in the without-bleeding event group.

OR, odds ratio = Exp (β); CI, confidence interval; OAC, oral anticoagulant
